# Mathematical Modeling of Hydroxyurea Therapy in Individuals with Sickle Cell Disease

**DOI:** 10.3390/pharmaceutics14051065

**Published:** 2022-05-16

**Authors:** Akancha Pandey, Jeremie H. Estepp, Rubesh Raja, Guolian Kang, Doraiswami Ramkrishna

**Affiliations:** 1 Davidson School of Chemical Engineering, Purdue University, West Lafayette, IN 47907, USA; pandey12@purdue.edu (A.P.); raja11@purdue.edu (R.R.); 2 Departments of Global Pediatric Medicine and Hematology, St. Jude Children’s Research Hospital, Memphis, TN 38105, USA; jeremie.estepp@stjude.org; 3 Department of Biostatistics, St. Jude Children’s Research Hospital, Memphis, TN 38105, USA; guolian.kang@stjude.org

**Keywords:** sickle cell disease, hydroxyurea, PK-PD, fetal hemoglobin, mean cell volume

## Abstract

Sickle cell disease (SCD) is a chronic hemolytic anemia affecting millions worldwide with acute and chronic clinical manifestations and early mortality. While hydroxyurea (HU) and other treatment strategies managed to ameliorate disease severity, high inter-individual variability in clinical response and a lack of an ability to predict those variations need to be addressed to maximize the clinical efficacy of HU. We developed pharmacokinetics (PK) and pharmacodynamics (PD) models to study the dosing, efficacy, toxicity, and clinical response of HU treatment in more than eighty children with SCD. The clinical PK parameters were used to model the HU plasma concentration for a 24 h period, and the estimated daily average HU plasma concentration was used as an input to our PD models with approximately 1 to 9 years of data connecting drug exposure with drug response. We modeled the biomarkers mean cell volume and fetal hemoglobin to study treatment efficacy. For myelosuppression, we modeled red blood cells and absolute neutrophil count. Our models provided excellent fits for individuals with known or correctly inferred adherence. Our models can be used to determine the optimal dosing regimens and study the effect of non-adherence on HU-treated individuals.

## 1. Introduction

Sickle cell disease (SCD) is a hereditary disorder caused by a single gene mutation in the β-globin gene that produces sickle hemoglobin (HbS) [1]. HbS polymerizes when deoxygenated and is the nidus for the complex downstream pathobiology observed in individuals with SCD, including acute SCD-related complications (vaso-occlusive pain, acute chest syndrome, priapism, etc.) and the onset and progression of end-organ damage [2]. SCD affects approximately 100,000 people in the United States and millions globally, and every year an estimated 300,000 children are born with sickle cell anemia (SCA) across the globe [3,4]. Hydroxyurea (HU) is approved by the Food and Drug Administration for adults and children aged 2–18 years with SCD, but it is widely utilized in children beginning as early as 9 months of age [5,6]. Although HU has multiple therapeutic benefits in individuals with SCD, the primary benefits are through increasing fetal hemoglobin (HbF) and additionally increasing mean cell volume (MCV) and reducing absolute neutrophil count (ANC) and total white blood cell (WBC) counts. Clinically, HU reduces the frequency of vaso-occlusive pain crises, acute chest syndrome, number of transfusions required, and total hospitalizations [7,8,9,10]. However, there are challenges associated with HU treatment: significant interpatient variability in PK-PD, the need for timely prediction of the optimal dose, and low rates of adherence [11,12]. The first challenge is addressed in this work through PK-PD model formulation. The successful formulation of PK-PD models allows the following things: the model can be used to test for various dosing regimens, incorporate non-adherence, study drug–drug interactions, and analyze the synergistic effect of the drug with other treatment methods.

Substantial inter-individual variability (IIV) has been reported in the PK of HU in several cohorts with a large coefficient of variation in clinical PK parameters, such as 49% for AUC and 39% for C_max_ in children in one study [13,14,15]. The hematologic response to HU treatment varies widely across individuals with SCD [16,17,18]. The variation observed could be partially due to variation in PK [19] and PD. Additionally, genetic polymorphisms may contribute to the variation observed in treatment response for a given exposure. Single nucleotide polymorphisms (SNPs) in the SAR1A promoter have been associated with variation in the HbF response of HU-treated individuals [20,21]. Other studies indicated the role of polymorphisms in genes regulating HbF production, HU metabolizing enzymes, and erythroid progenitor proliferation in the varying treatment responses of HU [22,23,24].

Mathematical models of HU have focused on modeling PK with a one or two-compartment model where a first-order absorption and first-order or metabolic or both elimination were used to describe the drug dynamics in the plasma [25,26,27]. Previous studies have performed population modeling of HU to model the individual subject behavior besides retaining the average population behavior using non-linear mixed effect models (NLME) [15,28,29,30]. These studies have identified weight as the significant covariate. Paule et al. also attempted to model the HbF and MCV dynamics using indirect response models [28]. Most of these studies have successfully modeled the PK without including PD.

The half-life of HU is 2–6 h, but the drug’s effect is seen on a timescale of weeks and takes months to stabilize [18]. Previous mathematical models of HU have focused on understanding the drug PK individually and on a population level in individuals with SCD [28,29]. There have been a few attempts to model the long-term effect of HU on the dynamics of hematologic parameters (MCV, Hb, HbF, etc.) and mathematically link drug exposure with drug response [28]. In addition, no toxicity model has been considered, which is important for identifying the optimal daily dose of HU. Recently, a PK-guided dosing strategy was employed to reduce the time to reach the maximum tolerated dose (MTD) to 4.8 months, starting at an average higher dose of 27.7 mg/kg/day [31] without showing hematological toxicity. However, this PK-based dosing strategy did not incorporate the role of PD variables in dose determination. It is imperative to include the PD model, which can capture the long-term cumulative effect of the drug and explain why the change in HbF and MCV is slow compared to the change in drug concentration.

We developed a mathematical model of HU that captures individuals with SCD PK-PD trajectories over longitudinal follow-up. The PK model is linked with the PD model to capture treatment efficacy and adverse effect along with drug kinetics. The PD model describes HU biomarker trajectories with a treatment period varying from less than 1 year to 9 years. The treatment efficacy is characterized by the HbF and MCV of red blood cells (RBC), both of which increase with HU treatment. The potential adverse effects of HU are myelosuppression characterized by reductions in RBC and ANC.

## 2. Materials and Methods

### 2.1. Clinical Data

#### 2.1.1. Observations from the Data

**Figure 1:**
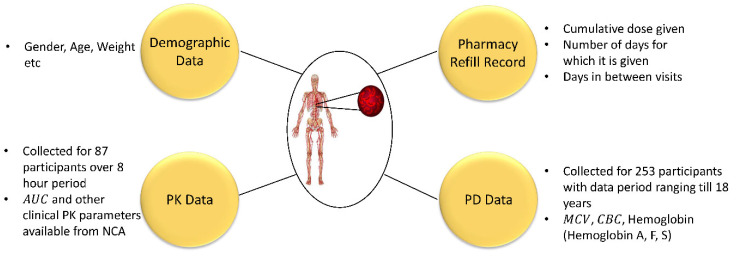
Description of HUSTLE participants’ data components.

We retrospectively analyzed data from the HUSTLE trial (NCT00305175) that were collected at St. Jude Children’s Research Hospital to study the long-term effects of HU therapy in children with SCD. The data contained participants’ demographics, PK, PD, and pharmacy refill records, as shown in Figure 1. The demographic data consisted of participants’ gender, age, weight, height, etc. The number of males to females was 54 to 31. At the start of treatment, the subjects’ ages ranged from 1.29 to 17.95 years old. The PK data were collected for 87 participants over 8 h at the beginning of HU treatment. The PK data did not include the exact plasma drug concentration versus time data. Instead, the PK data consisted of the AUC and other clinical PK parameters available from non-compartmental analysis (NCA). The PD data were collected for a larger cohort of 253 participants, with the data collection period ranging from less than 1 to 18 years across the population. The data consisted of complete blood count (CBC), MCV, and hemoglobin fractions via high-performance liquid chromatography such as hemoglobin A, F, and S versus time. Table 1 lists the summary of participants with SCD demographics, clinical PK parameters, and laboratory values of PD variables. The laboratory values were reported at the start (±10 days), after 6 (±1) months, and after 12 (±1) months of HU treatment. The pharmacy refill record provided the total dose given, the number of days for which it was given, and the days between participant visits. If the number of days for which the capsules were given was less than the number of days between participant visits, it indicated a clear case of non-adherence.

#### 2.1.2. Data for Modeling

PK and PD data were available for 87 participants with SCD who were prescribed HU, and 85 participants’ data were used to construct the model. Two participants were not included in the model because one had only a single data point, and one had no pharmacy data. Among the clinical variables, the key variables for modeling included MCV and HbF, biomarkers to indicate treatment efficacy. Additionally, to capture myelosuppression, the RBC and ANC profiles were modeled. Of particular interest to us was the ANC, as it helps clinicians decide the maximum tolerated dose (MTD) of HU administered. The MTD is determined when the ANC reaches the target range between 2000–4000 cells/µL.

**Figure 2:**
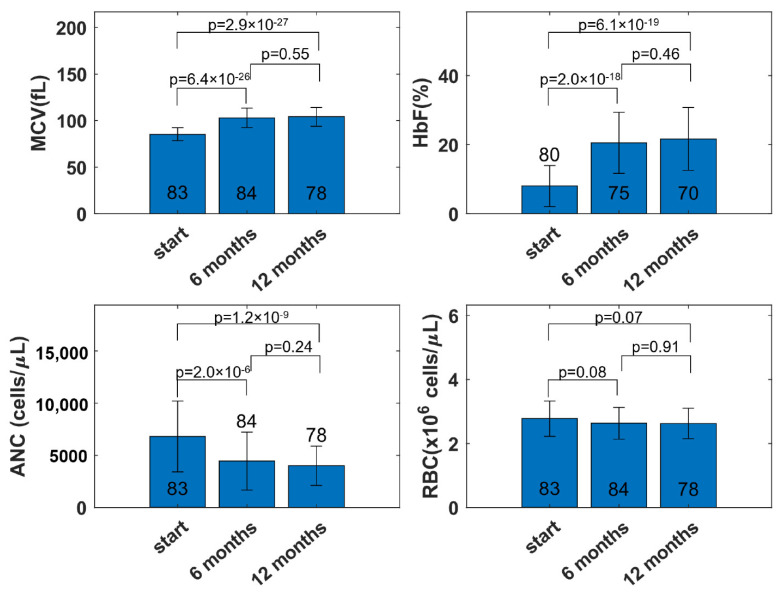
Trends in clinical variables averaged across participants at the start and following 6 months and 12 months of hydroxyurea treatment. The bar plot represents the mean; the error bar represents the standard deviation of the clinical variables; the number on the bar plot represents the number of individuals. MCV, mean cell volume; HbF, fetal hemoglobin; RBC, red blood cell; ANC, absolute neutrophil count.

In Figure 2, the average values and trends for the key variables of interest with HU treatment are seen over the course of 1 year of treatment. The two biomarkers, HbF and MCV, increased with the onset of HU treatment until 6 months, stabilizing following 6 months of treatment. The number of data points for HbF was lower than the number of data points for MCV, RBC, and ANC. As part of standard medical care, HbF was not collected at each visit. It was observed that some participants experienced decreases in MCV and HbF over time after 1 year of therapy, potentially due to non-adherence.

The ANC and WBC of individuals with SCD are elevated without therapy [32,33]. Hydroxyurea normalizes the average ANC and WBC. The average ANC decreased from ~7000 cells/µL at the beginning to the desired level of 2000–4000 cells/µL after 6 months of HU treatment and remained stable afterward. The ANC and WBC (not shown in the figure) fluctuated for some participants, potentially due to changes in the drug amount, non-adherence, and several other reasons, such as common viral infections. With RBC, two factors are in play when HU is administered: (i) the drug decreases RBC due to myelosuppression, and (ii) the drug increases RBC due to reduced hemolysis, increasing the lifespan of RBC [34]. As a result, the average RBC did not fluctuate and remained stable at around 2.5 million cells/µL. The individual participant RBCs fluctuated within a constant range for some participants, while the myelosuppression effect was dominant for others.

### 2.2. Modeling

**Figure 3:**
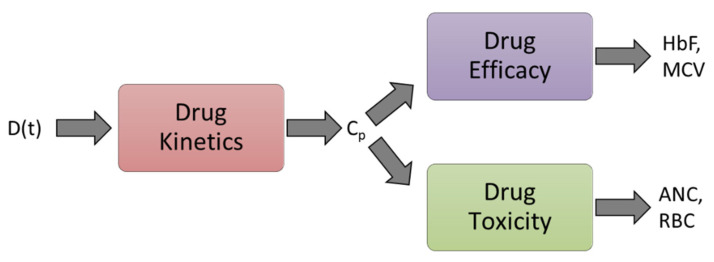
Integrated PK-PD model components. D(t), dose; $C_{p}$, HU plasma conc.; HbF, fetal hemoglobin; MCV, mean cell volume of red blood cell; ANC, absolute neutrophil count; RBC, red blood cell.

The different model components include modeling drug kinetics (PK) that describes how the drug gets absorbed, distributed, metabolized, and excreted from the body. For the PK model, the input is the drug dose, D, and the output is the drug concentration in the plasma, $C_{p}$. The second component includes modeling drug efficacy, which is captured by HbF and MCV dynamics. The efficacy model describes how the HbF and MCV levels change with respect to changes in $C_{p}$. The third component includes modeling drug safety/toxicity, captured by how the blood cells such as ANC and RBC counts change against $C_{p}$. Figure 3 shows the integrated PK-PD model components. Data analysis and modeling were performed in MATLAB R2020b [35]. HU is found to activate HbF through cellular signaling pathways [36,37,38]. For modeling the effect of HU on HbF on a cellular level, the mean cell fetal hemoglobin, $F_{m}$, was calculated as shown in Appendix A. While calculating $F_{m}$, the assumption was that HbF was uniformly distributed across all RBCs.

#### 2.2.1. Dose Calculation

**Figure 4:**
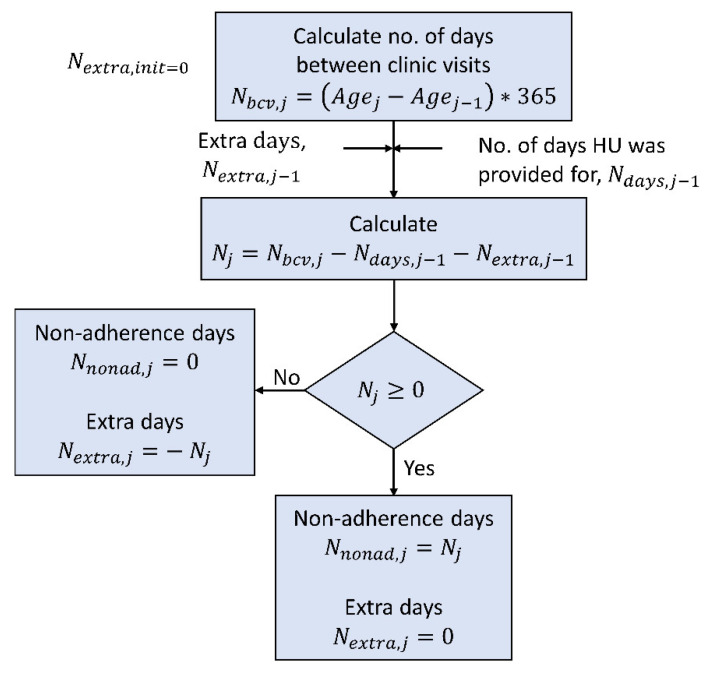
Flowchart to calculate the number of non-adherent days for jth visit from the pharmacy refill record. The pharmacy refill record contains the total dose given to the participant at every visit and the number of days for which it is given. This process assumes that the participant takes the capsule if they have it. $Age_{j-1}$ and $Age_{j}$ —age in years at j−1th and jth visit; $N_{bcv,j}$ —number of days between clinic visits; $N_{days,j-1}$ —number of days HU was provided at j−1th visit; $N_{extra,j}$ —number of extra days for which capsules are available at jth visit; $N_{extra,init}$ —number of initial extra capsules is assumed to be zero; $N_{nonad,j}$ —number of non-adherence days at jth visit.

The challenge with dose calculation was that the dosing information was not provided when the participant laboratory samples were collected. The dosing information was obtained from the pharmacy refill records, which listed the total dose provided, the age at which the dosing was given, and the number of days for which the drug was given. The number of days between clinic visits, $N_{bcv,j}$ at jth visit, was computed by subtracting the participant’s age between two consecutive visits, as shown in Figure 4. If $N_{bcv,j}$ exceeded the number of days for which the drug was provided, $N_{days,j-1}$, the assumption was that the participant was consuming any extra capsules remaining from j−1th visit, $N_{extra,j-1}$. Then, the number of days for which there was no capsule from the current, j or prior, j−1 visits was calculated to compute the number of missed days between clinic visits, $N_{nonad,j}$. Therefore, the number of missed and extra doses was calculated by subtracting $N_{bcv,j}$ from $N_{days,j-1}$ and $N_{extra,j-1}$ as shown in the flowchart. With dose calculation, the primary assumption was that if the participant had the capsule available, they consumed it; otherwise, the dose was missed only due to lack of availability of the capsule. Suppose there were extra capsules accumulated from previous times. In that case, the assumption was that the participant used it later.

The daily dose was assumed to be constant between visits and calculated in mg/kg by dividing the total dose by participants’ changing weight. The weight of participants was measured at every visit and was calculated by interpolation for in-between visits. Once $N_{nonad,j}$ was determined, the non-adherent days were selected randomly from $N_{bcv,j}$. The everyday dose was plugged into the PK model to obtain the $C_{p}$ versus time profile.

#### 2.2.2. Pharmacokinetic Model

**Figure A1:**
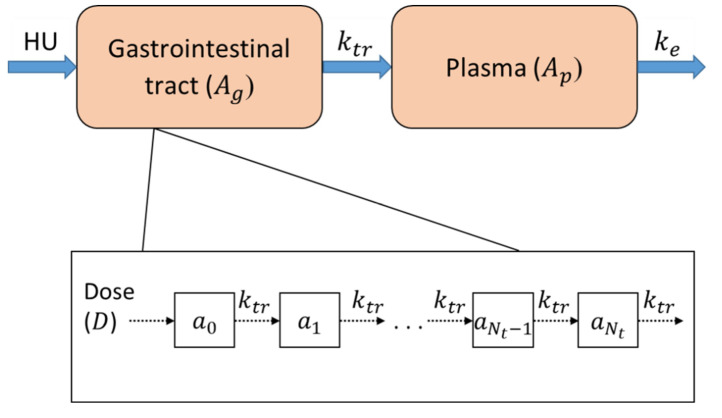
Schematic of hydroxyurea pharmacokinetic model.

The PK model consists of two compartments, a gastrointestinal (GI) tract and a plasma compartment (Figure A1). Hydroxyurea is taken orally. It travels through the GI tract and is absorbed in the plasma with the first-order rate constant, $k_{tr}$. From plasma, HU is eliminated either via renal or metabolic pathways with the first-order rate constant, $k_{e}$. To capture the different absorption profiles, as observed in individuals with SCD taking HU, a transit compartment model for absorption is considered, consisting of a series of compartments to introduce an exponential delay term [29,39]. It can adequately describe rapid or delayed absorption by varying the number of transit compartments. The rate of change in the amount of HU in the plasma compartment, $A_{p}$, is given by,

(1) $$\frac{dA_{p}}{d_{t}}=k_{tr}a_{Nt}-k_{e}A_{p}$$

where $\left(N_{t}+1\right)$ is the number of transit compartments, $a_{N_{t}}$ is the drug amount in the final transit compartment in the gut calculated using Equation (A4) in Appendix A [29,39].

The volume of plasma, $V_{p}$ is obtained by using the following formula,

(2) $$V_{p}=V_{b}\left(1-\frac{\mathrm{HCT}}{100}\right)$$

where $V_{b}$ is the volume of blood obtained using an empirical formula. For subjects’ weight ≥ 25 kg, the $V_{b}$ is obtained using the Nadler equation given below [40]:

(3) $$\begin{array}{c} \mathrm{Male}:\;V_{b}\left(L\right)=0.3669\;\mathrm{height}\;{\left(\mathrm{cm}\right)}^{3}+0.03219\;\mathrm{weight}\;\left(\mathrm{kg}\right)+0.6041\\ \mathrm{Female}:\;V_{b}\left(L\right)=0.3561\;\mathrm{height}\;{\left(\mathrm{cm}\right)}^{3}+0.03308\;\mathrm{weight}\;\left(\mathrm{kg}\right)+0.1833 \end{array}$$

For subjects’ weight < 25 kg, the $V_{b}$ is scaled by 70 mL/kg as shown below [41,42]:

(4) $$V_{b}\left(L\right)=\frac{70}{1000}\left(\frac{L}{\mathrm{kg}}\right)\mathrm{weight}\;\left(\mathrm{kg}\right)$$

The drug concentration in the plasma, $C_{p}$ is obtained by the following equation:

(5) $$C_{p}=\frac{A_{p}}{V_{p}}$$

#### 2.2.3. Parameter Estimation for PK Model

The parameter estimation for the PK model started with an initial guess for the PK parameters. The PK data provided did not consist of the time course of measured $C_{p}$ data points. Instead, it consisted of clinical PK parameters obtained from NCA. The clinical PK parameters in the data consisted of AUC, AUC_∞_, MRT_∞_, T_max_, C_max_, and λ_z_. The area under the first moment of the concentration–time curve extrapolating to ∞ is obtained by the following formula:

(6) $${\mathrm{AUMC}}_{\infty }={\mathrm{MRT}}_{\infty }\times {\;\mathrm{AUC}}_{\infty }$$

The clinical PK parameters were calculated from the $C_{p}$ versus t plot obtained from the model. Ware et al. [14] measured HU concentrations in plasma at the following time points: *t* = 0, 15 min, 30 min, 1, 2, 4, 6, and 8 h after drug administration. The last time the measurements were made was 8 h after HU administration. So, 8 h was used as the last time point to obtain AUC, and 24 h was used as the time point to obtain ${\mathrm{AUC}}_{\infty }$, ${\mathrm{AUMC}}_{\infty }$. The AUC, ${\mathrm{AUC}}_{\infty }$, and ${\mathrm{AUMC}}_{\infty }$, from the model were calculated by the following equation:

(7) $$\mathrm{AUC}=\int_{0}^{t_{last}}C_{p}\left(t\right)dt;\;{\mathrm{AUC}}_{\infty }=\int_{0}^{24}C_{p}\left(t\right)dt;\;{\mathrm{AUMC}}_{\infty }=\int_{0}^{24}tC_{p}\left(t\right)dt$$

The maximum concentration, $C_{\max}$, and the time at which the drug reaches the peak value, $T_{\max}$, were calculated from the model. The rate constant of elimination, $k_{e}$, was considered to be the same as $\lambda_{z}$. The four model parameters, F, N, $k_{tr}$, and $k_{e}$, for every subject were calculated by minimizing the weighted sum of square error given below:

(8) $$\underset{\theta }{\min}\overset{6}{\underset{j=1}{\sum }}{\left(\frac{{\hat{y}}_{j}\left(\theta \right)-y_{j,\;clinical\;data}}{w_{j}}\right)}^{2}$$

where $y_{j,clinical\;data}$ is the clinical data value for the jth clinical PK parameter consisting of AUC, ${\mathrm{AUC}}_{\infty }$, ${\mathrm{AUMC}}_{\infty }$, $T_{\max}$, $C_{\max}$, and $\lambda_{z}$. ${\hat{y}}_{j}\left(\theta \right)$ is the model prediction for the jth clinical PK parameter, θ is the set of PK model parameters, $w_{j}$ is the weight associated with jth clinical data. Further, the two metrics were used as weights in the cost function shown in Equation (8). The first was when the individual data points for every clinical PK parameter were used as weights, and the second was when the means of every clinical PK parameter across all participants were used as weights. The means of every clinical PK parameter as weights produced better fits. The model was implemented in MATLAB R2020b [35] using MultiStart optimization algorithm to estimate θ. Once the PK model parameters were estimated, the $C_{p}$ versus t plot was obtained, from which the daily average $C_{p}$, ${\bar{C}}_{p}$ were calculated as shown in Appendix A (Equation (A5)). The PK model simulations were performed every day with dose as input, and the ${\bar{C}}_{p}$ was computed for every day. The ${\bar{C}}_{p}$ was then taken as the input for the PD models, which included modeling the effect of HU on erythropoiesis, leukopoiesis process, and HbF activation by HU. The erythropoiesis and leukopoiesis processes were modeled because HU targets the actively dividing cells present in the bone marrow in the initial stages of erythropoiesis and leukopoiesis, which eventually manifest in the cells in circulation [43].

#### 2.2.4. Erythropoiesis and MCV Model

**Figure 5:**
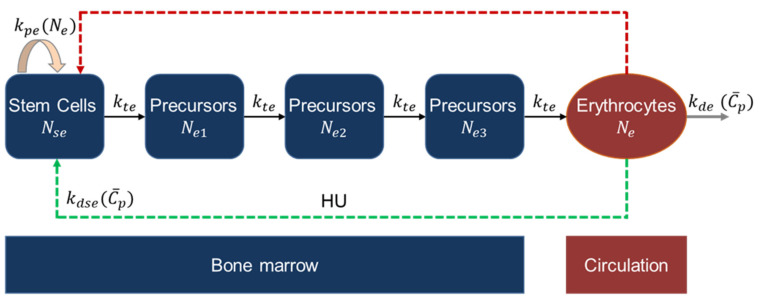
Schematic of erythropoiesis model. The dashed red arrow represents negative feedback regulation from the circulating cells; the dashed green arrow represents HU-related effect.

The erythropoiesis and MCV models to study the effect of HU on RBC and MCV were adapted from the work of Jayachandran et al., 2014 [44]. The erythropoiesis model divides the cells into five compartments, as shown in Figure 5. The stem cell compartment denoted as $N_{se}$, consists of stem cells and early proliferating progenitors, which proliferate at the rate $k_{pe}$. The proliferation is regulated by a cytokine, erythropoietin (EPO), whose production is regulated by RBCs in the periphery [45]. In SCD, RBCs undergo hemoglobin polymerization and hemolysis, resulting in decreased oxygen delivery to the cells and tissues. The hypoxia induces EPO production in the kidney, which upregulates erythroid progenitors [46]. The indirect effect of circulating cells on progenitors’ proliferation is modeled here without incorporating the EPO expression. It is assumed that HU targets only proliferating cells in the $N_{se}$ compartment. The cells from the $N_{se}$ compartment transition and go through three precursor compartments where cells do not undergo proliferation but only maturation. The precursor compartments are denoted as $N_{e1}$,$\;N_{e2}$, $N_{e3}$. Finally, the precursor cells become fully functional erythrocytes, denoted as $N_{e}$. The erythrocytes or RBCs circulate in the body for ~120 days [47] and die at a rate dependent on the drug. The death rate is modeled as a function of HU because the erythrocyte half-life is dependent on HU exposure. This is due to HU increasing the lifespan of RBCs in addition to being myelosuppressive to stem cells and progenitors [34]. The model equations are given in Equation (9).

(9) $$\begin{array}{c} \frac{dN_{se}}{dt}=k_{pe}\left(N_{e}\right)-k_{te}N_{se}-k_{dse}\left({\bar{C}}_{p}\right)N_{se}\\ \frac{dN_{e1}}{dt}=k_{te}N_{se}-k_{te}N_{e1}\\ \frac{dN_{e2}}{dt}=k_{te}N_{e1}-k_{te}N_{e2}\\ \frac{dN_{e3}}{dt}=k_{te}N_{e2}-k_{te}N_{e3}\\ \frac{dN_{e}}{dt}=k_{te}N_{e3}-k_{de}\left({\bar{C}}_{p}\right)N_{e} \end{array}$$

To avoid complexity, the RBC-controlled EPO production and EPO-controlled progenitors’ proliferation are bypassed, and the effect of RBCs on proliferation rate $k_{pe}$ is directly modeled through a negative feedback mechanism; $k_{pe}$ is negatively correlated to RBCs and is modeled using Hill kinetics.

(10) $$k_{pe}\left(N_{e}\right)=k_{pe,max}\frac{\Psi_{e}{}^{\gamma_{e}}}{\left(\Psi_{e}{}^{\gamma_{e}}+N_{e}{}^{\gamma_{e}}\right)}$$

where $k_{pe,max}$ is the maximum proliferation rate, $\gamma_{e}$ is the steepness parameter for feedback, $\Psi_{e}$ is the feedback parameter. To model myelosuppression in the $N_{se}$ compartment by HU, the model has a death rate constant, $k_{dse}$, which is dependent on $C_{p}$ and is modeled using Hill-type kinetics as shown below:

(11) $$k_{dse}\left({\bar{C}}_{p}\right)=k_{dse,max}\frac{{\bar{C}}_{p}}{K_{dse,50}+{\bar{C}}_{p}}$$

where $k_{dse,max}$ is the maximum death rate constant due to HU, $K_{dse,50}$ is the saturation constant for the effect of HU on RBC. The cells are transferred from the stem cell to precursor to erythrocyte compartments at a rate constant, $k_{te}$. Further, the death rate of RBC is assumed to be dependent on $C_{p}$ to model the increased lifespan of RBC due to HU. The death rate constant $k_{de}$ for RBC is modeled as shown below:

(12) $$k_{de}=k_{de,max}\left(1-\frac{{\bar{C}}_{p}}{K_{de,50}+{\bar{C}}_{p}}\right)$$

where $k_{de,max}$ is the maximum death rate constant for RBC, $K_{de,50}$ is the saturation constant for the drug.

##### MCV Model

MCV is used as a biomarker to indicate treatment efficacy. The MCV model was adapted from the work of Jayachandran et al. [44]. MCV is obtained by dividing the total volume of RBCs by the total count of RBCs, assuming every RBC has the same volume. The total volume of cells in the circulation increases due to the influx of cells from the precursor compartments in the bone marrow and the HU-induced increase in MCV. These cells have baseline MCV, $V_{m0}$, and there is an increase in MCV due to HU. The increase in MCV due to HU is assumed to be a linear function of drug concentration, $\alpha {\bar{C}}_{p}$. The total volume of cells decreases due to the death of RBCs with the current MCV, $V_{m}$. The rate of change in total volume of all the RBCs, $V_{TOT}$, is given by,

(13) $$\frac{dV_{TOT}}{dt}=\left(\alpha {\bar{C}}_{p}+V_{m0}\right)k_{te}N_{e3}-V_{m}k_{de}N_{e}$$

The MCV is derived in Appendix A and given by the following formula:

(14) $$\frac{dV_{m}}{dt}=\frac{\left(\alpha {\bar{C}}_{p}+V_{m0}-V_{m}\right)k_{te}N_{e3}}{N_{e}}$$

#### 2.2.5. Leukopoiesis Model

**Figure 6:**
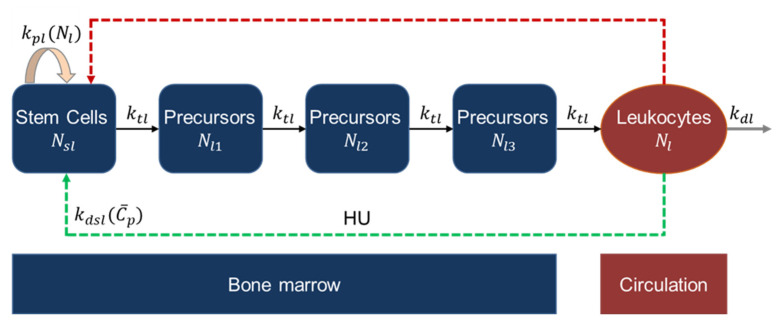
Schematic of leukopoiesis model. The dashed red arrow represents negative feedback regulation from the circulating cells; the dashed green arrow represents HU-related effect.

The leukopoiesis process produces leukocytes that play an essential role in defending the body against foreign invasions and inflammation [48]. The process was modeled to study the effect of HU on the progenitors and precursor cells, and eventually the leukocytes. A leukopoiesis model similar to the erythropoiesis model was adapted from the work of Jayachandran et al. [44], and the schematic is shown in Figure 6. The stem cells and proliferating cells are represented as $N_{sl}$. The neutrophil precursors are represented as cells in three precursor compartments denoted as $N_{l1}$, $N_{l2}$, $N_{l3}$. The ANC in the circulation is represented by the cells in the final compartment, $N_{l}$. The model equations are shown below:

(15) $$\begin{array}{c} \frac{dN_{sl}}{dt}=k_{pl}\left(N_{l}\right)-k_{tl}N_{sl}-k_{dsl}\left({\bar{C}}_{p}\right)N_{sl}\\ \frac{dN_{l1}}{dt}=k_{tl}N_{sl}-k_{tl}N_{l1}\\ \frac{dN_{l2}}{dt}=k_{tl}N_{l1}-k_{tl}N_{l2}\\ \frac{dN_{l3}}{dt}=k_{tl}N_{l2}-k_{tl}N_{l3}\\ \frac{dN_{l}}{dt}=k_{tl}N_{l3}-k_{dl}N_{l} \end{array}$$

The proliferation of leukocytes is regulated by a cytokine, granulocyte-macrophage colony-stimulating factor (GM-CSF) [49]. The proliferation rate, $k_{pl}$, is inversely proportional to neutrophil count and is given by the following equation:

(16) $$k_{pl}\left(N_{l}\right)=k_{pl,max}\frac{{\Psi_{l}}^{\gamma_{l}}}{\left({\Psi_{l}}^{\gamma_{l}}+{N_{l}}^{\gamma_{l}}\right)}$$

where $k_{pl,max}$ is the maximum proliferation rate constant, $\gamma_{l}$ is the steepness parameter for feedback, $\Psi_{l}$ is the feedback parameter. HU targets the cells in the stem cell compartment, and the death rate constant, $k_{dsl}$, is modeled by Hill-type kinetics, as shown below:

(17) $$k_{dsl}\left({\bar{C}}_{p}\right)=k_{dsl,max}\frac{{\bar{C}}_{p}}{K_{dsl,50}+{\bar{C}}_{p}}$$

where $k_{dsl,max}$ is the maximum death rate constant, $K_{dsl,50}$ is the saturation constant for the effect of HU on ANC.

#### 2.2.6. Fetal Hemoglobin Model

**Figure 7:**
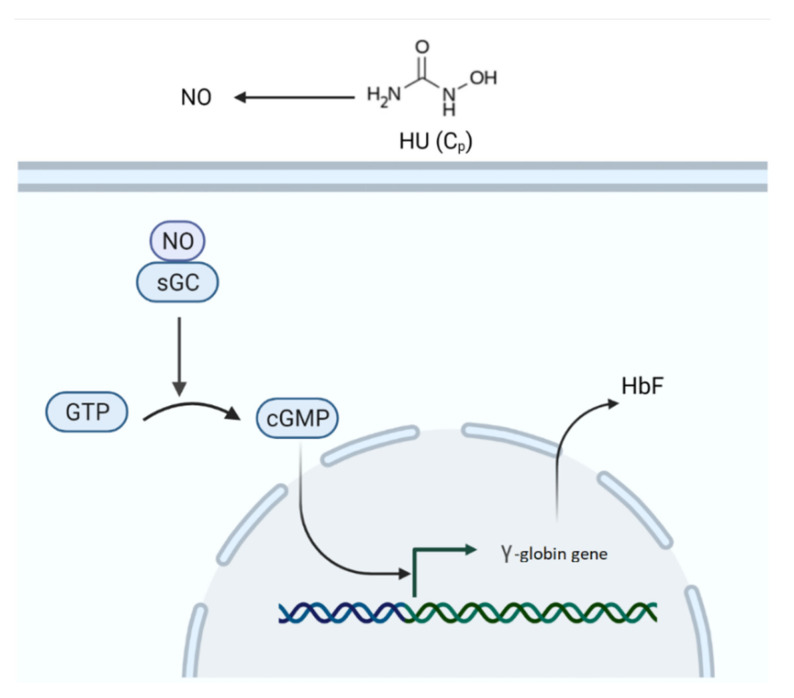
Hydroxyurea-mediated activation of fetal hemoglobin. HU, hydroxyurea; NO, nitric oxide; sGC, soluble guanylyl cyclase; GTP, guanosine triphosphate; cGMP, cyclic guanosine monophosphate; HbF, fetal hemoglobin.

For HbF, the formulated model captures its production in an average RBC due to HU. The assumption here is that every RBC makes the same amount of HbF. The HbF% is highest at birth and decreases rapidly until 4–6 months after birth, after which it diminishes gradually and reaches a minimum level after a year [50]. Some individuals have unusually high HbF levels even after 1 year of age due to hereditary persistence of fetal hemoglobin (HPFH), which protects against SCD symptoms [51,52]. The individuals with HPFH condition express elevated HbF levels in the range of 10–40% [53]. The high expression of HbF level in some individuals was correlated to their haplotype [54]. The baseline HbF varied between 0–28% in the HUSTLE data. Therefore, the model includes a basal rate of production of HbF, which is independent of HU to account for subjects’ inherent machinery for the production of HbF that might vary with subjects’ age. Studies showed that HU metabolizes into nitric oxide (NO) and its derivatives, such as hydroxylamine, urea, nitrite, and nitrate [55,56]. The NO binds to soluble guanylate cyclase (sGC) inside the cell and activates it [36,37]. The activated sGC is known to convert guanosine triphosphate (GTP) to cyclic guanosine monophosphate (cGMP). Studies suggested the role of cGMP in HU-induced activation of HbF [36,37], as shown in Figure 7.

Without going into the complexities of signaling pathways, only two components are modeled here. One is intermediate produced from HU, and the other is HbF. The exact intermediates of HU are not known, and all the possible intermediates are clubbed into one. With this hypothesis, the rates of change in intermediates and HbF with time are modeled. In the model, HU is metabolized to an intermediate represented as $C_{i}$. This intermediate could be NO or its derivatives. The $C_{i}$ production from HU happens through Michaelis–Menten kinetics owing to the involvement of enzymes in the degradation of HU into NO [57,58], and $C_{i}$ is degraded (Equation (18)). The first term in the HbF equation denotes the inherent or basal rate of production of HbF, $k_{bf}$, in the absence of HU (Equation (19)). The second term represents the activated rate of production of HbF in the presence of HU through the intermediate $C_{i}$ and is modeled using Hill kinetics. The third term denotes the degradation of HbF. The model equation is shown below:

(18) $$\frac{dC_{i}}{dt}=k_{met}\frac{{\bar{C}}_{p}}{K_{met}+{\bar{C}}_{p}}-k_{di}C_{i}$$

(19) $$\frac{dF_{m}}{dt}=kb_{f}+ka_{f}\frac{{C_{i}}^{n}}{\left({ka_{f}}^{n}+{C_{i}}^{n}\right)}-kd_{f}F_{m}$$

where $C_{i}$ is the intermediate concentration, $k_{met}$ is the maximum rate constant for the intermediate production from HU, $K_{met}$ is the Michaelis constant, $k_{di}$ is the degradation rate constant for $C_{i}$, $k_{bf}$ is the basal rate of production of HbF, $k_{af}$ is the maximum rate of $C_{i}$-induced HbF activation, n is the Hill coefficient, $K_{af}$ is the half-saturation constant, $k_{df}$ is the degradation constant for HbF.

#### 2.2.7. Parameter Estimation

For the parameter estimation, multiple methods, including non-linear least-squares solver and derivative-free search, were run in series. A combination of functions lsqnonlin, fmincon, patternsearch was used from MATLAB R2020b [35], and the model was solved 25 times starting from 25 initial guesses generated randomly from a uniform distribution. The final parameter set that gave the lowest cost function and with a visually good-looking fit was selected.

The cost function, which was minimized, is the weighted sum of square errors, as shown below:

(20) $$\underset{\theta }{\min}\overset{N_{var}}{\underset{j=1}{\sum }}\overset{N_{exp}}{\underset{i=1}{\sum }}{\left(\frac{\hat{y_{j}}\left(t_{i}|\theta \right)-y_{j}\left(t_{i}\right)}{w_{j}}\right)}^{2}$$

where subscript i represents the time index, j represents the clinical variable index. $y_{j}\left(t_{i}\right)$ is the jth clinical data at ith time point, $\hat{y_{j}}\left(t_{i}|\theta \right)$ is the model predicted jth clinical data at ith time point given model parameters, θ. Due to the optimization of more than one clinical variable, the cost function is normalized using weights, $w_{j}$. $N_{exp}$ is the total number of clinical time points, and $N_{var}$ is the total number of clinical variables. The bifurcation analysis was performed to determine the parameter bounds for the ANC model in XPPAUT [59].

## 3. Results

### 3.1. PK Model

**Figure 8:**
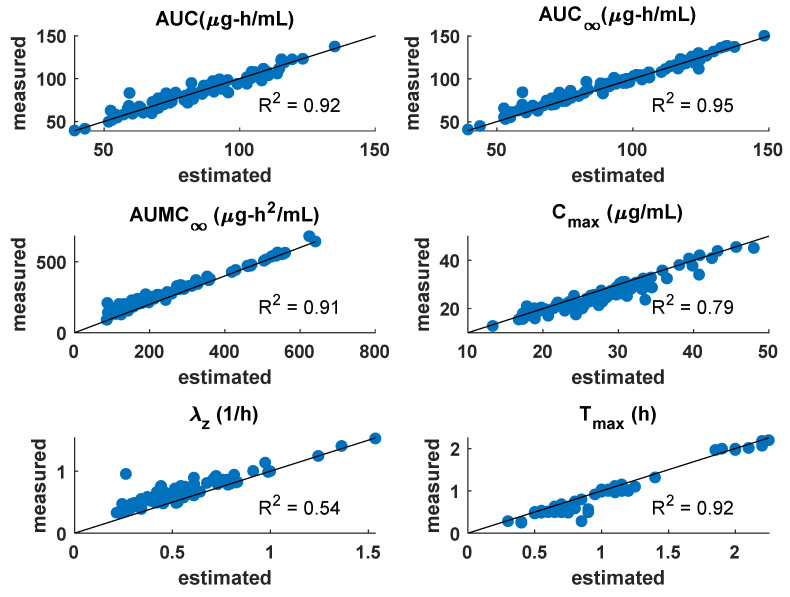
Goodness-of-fit plots for the PK model for the following clinical PK parameters: AUC, ${\mathrm{AUC}}_{\infty }$, ${\mathrm{AUMC}}_{\infty }$, $C_{\max}$, $\lambda_{z}$, $T_{\max}$.

In the PK model, the AUC, ${\mathrm{AUC}}_{\infty }$, ${\mathrm{AUMC}}_{\infty }$, $C_{\max}$, $\lambda_{z}$, $T_{\max}$ data from every participant were used to fit the individual PK model and estimate the PK parameters: F, $k_{tr}$, $N_{t}$, $k_{e}$. The goodness-of-fit plots are shown in Figure 8. It shows the measured versus model-estimated values for the clinical PK parameters of AUC, ${\mathrm{AUC}}_{\infty }$, ${\mathrm{AUMC}}_{\infty }$, $C_{\max}$, $\lambda_{z}$, $T_{\max}$, and the goodness-of-fit was measured by $R^{2}$. Each of the dots represents individual participant data that were modeled. For most of the clinical variables such as AUC, ${\mathrm{AUC}}_{\infty }$, ${\mathrm{AUMC}}_{\infty }$, $C_{\max}$, and $T_{\max}$, the model estimates matched well with the measured values of these variables, as seen from $R^{2}\geq 0.75$. The model could not predict well for $\lambda_{z}$ with $R^{2}=0.56$, as for some participants, the estimated $\lambda_{z}$ was less than the measured value. Further, Table 2 lists the statistics of estimated PK parameters. The average value of bioavailability, F, was 0.12, which was lower than the F value of 0.7, or higher as reported earlier in the HU studies conducted in cancer [25,27].

**Figure 9:**
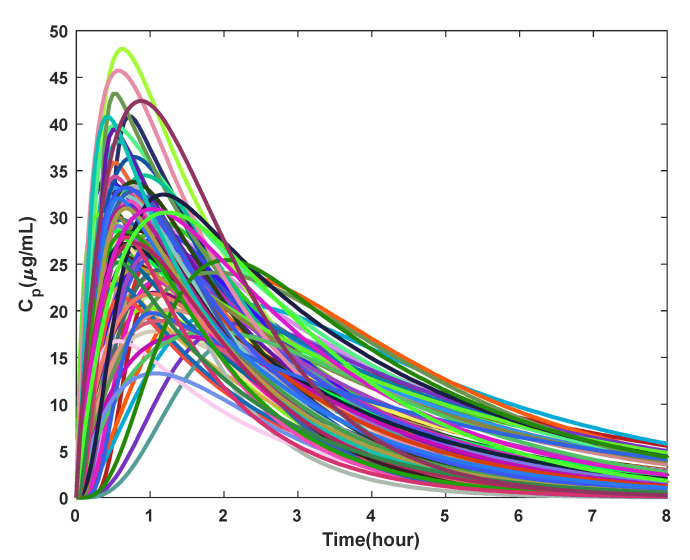
Pharmacokinetic plots for 87 participants with every color marking an individual participant.

Using the estimated PK model parameters, the $C_{p}$ versus time plot was obtained for 87 participants, as shown in Figure 9. The $C_{p}$ increased and reached its peak value in 1–2 h. The drug was cleared from the body within 24 h. The generated participant PK data resembled the true PK data from the HUSTLE study [14]. As shown in Ware et al. [14], participants with fast and slow absorption profiles were also seen from the PK plots generated here.

**Figure 10:**
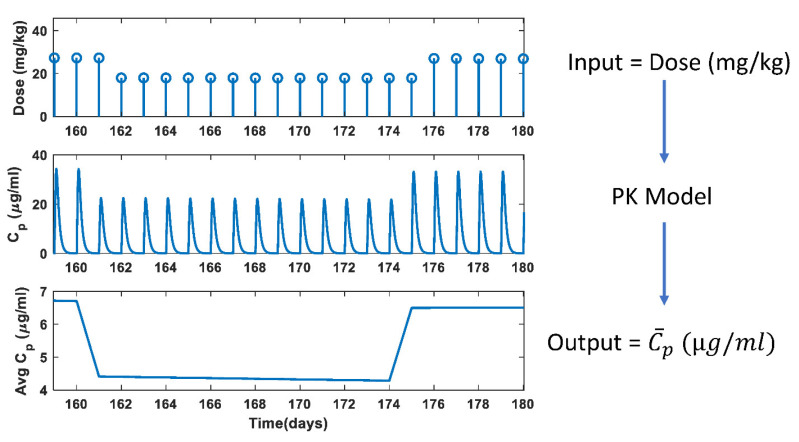
Pharmacokinetic model simulations performed every day for a representative participant.

Once the PK model parameters were estimated, the PK model was simulated daily with the dose calculated from the pharmacy data. Since the PK model gives $A_{p}$, it is divided by $V_{p}$ to obtain $C_{p}$. The $V_{p}$ was calculated daily by computing the participant’s weight, height, and HCT. The four PK model parameters remained constant with time, but the change in dose changed the $C_{max}$ and other clinical PK parameters. Figure 10 demonstrates PK model simulations performed every day for a representative participant with the daily dose as input. The top plot shows the everyday dose, and the middle plot shows the $C_{p}$ versus time plot where the peak concentration, $C_{max}$ changes with change in the drug input. The ${\bar{C}}_{p}$ was computed every day, and $C_{p}$ was assumed to be constant at ${\bar{C}}_{p}$ for the entire day, which was then plugged into the PD model to study the effect of change in drug input on the biomarker dynamics.

### 3.2. MCV and RBC Models

**Figure 11:**
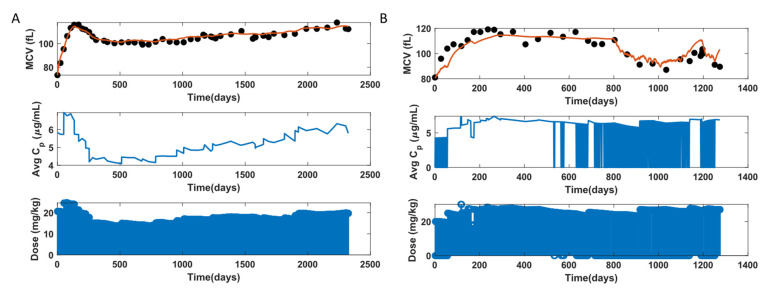
MCV model fit to data for two representative participants. (**A**): Adherent, (**B**): Non-adherent.

**Figure 12:**
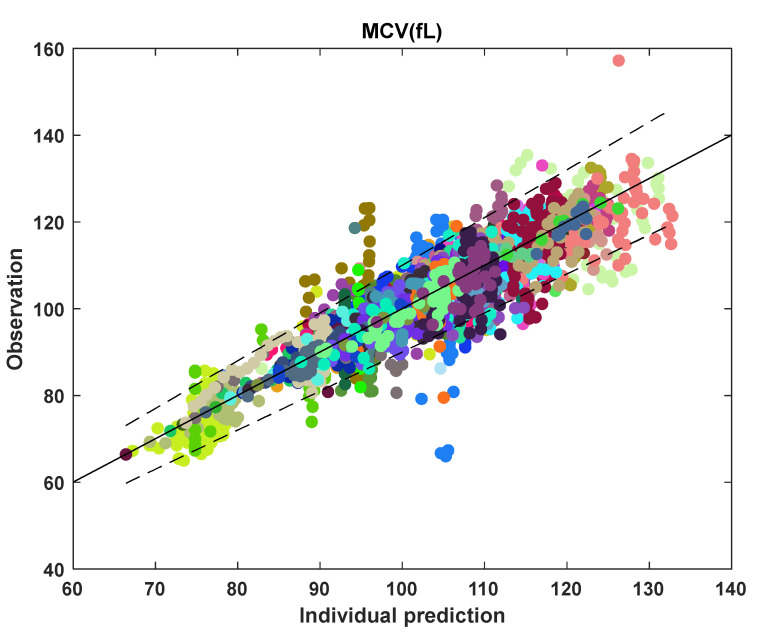
Goodness-of-fit plot for all MCV data points, where each color marks an individual participant. The solid line represents y = x; the dashed lines represent 10% error.

The MCV and RBC models were fit to the clinical data of 85 individuals. Initial conditions were chosen from the baseline values of the individual data. Figure 11A shows ${\bar{C}}_{p}$ plot in the middle, and the drug dose in mg/kg for every day in the bottom plot. The ${\bar{C}}_{p}$ plot shows an increase, followed by a decrease and then an increase again in the ${\bar{C}}_{p}$ value. This change in ${\bar{C}}_{p}$ is essentially a manifestation of change in drug input. The change in ${\bar{C}}_{p}$ is also reflected in the MCV behavior, because the MCV rises when ${\bar{C}}_{p}$ increases and then drops as ${\bar{C}}_{p}$ decreases, and so on. The model captures the dynamical changes in MCV with HU treatment initiation and with changes in ${\bar{C}}_{p}$ and fits well for this fully adherent participant. Figure 11B depicts a non-adherent participant as seen from the ${\bar{C}}_{p}$ profile for this participant. The regions of blue block in ${\bar{C}}_{p}$, for example, from 700 to 1000 days, indicates the presence of multiple non-adherent days. There is also a drop in the MCV data between 700 to 1000 days, indicating potential non-adherence. The model fits this drop in MCV due to non-adherence when the dosing profile contains the non-adherence information. On the other hand, the MCV data between 350 and 400 days suggest non-adherence, but the dosing profile, as seen from ${\bar{C}}_{p}$, does not contain non-adherence information. As a result, the model does not fit the drop in MCV in this region. Therefore, the model mimics adherent and non-adherent participant behavior subject to the condition that the dosing profile contains the non-adherence information. Figure 12 shows the observation versus individual prediction for all participants at all time points. The participants are color-coded, where each color represents an individual. This figure shows that the model fits well to data for most participants, and the data points fall within the 10% error of y = x line for ~95% of total MCV data.

**Figure 13:**
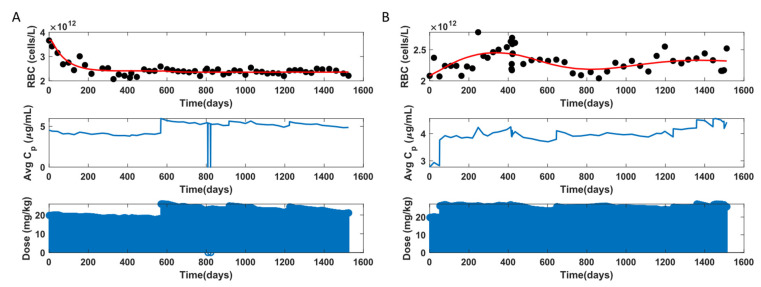
RBC model fit to data for two representative participants. (**A**): with myelosuppression, (**B**): without myelosuppression.

There are two broad categories of profiles observed for RBC data. In one category, the participant’s RBC decreases when HU treatment is started and stabilizes after some time. These individuals show a clear myelosuppression trend. Another category is where the RBC data fluctuate and lack a clear trend. These individuals do not show myelosuppression. So, when the RBC data indicate myelosuppression, the model mimics that trend, as seen in Figure 13A. When the RBC is fluctuating, the model is not able to capture all the points. The model fits fluctuating RBC data with a straight line or a curve, as seen in Figure 13B, where the model fit tries to pass through as many data points as possible.

**Figure 14:**
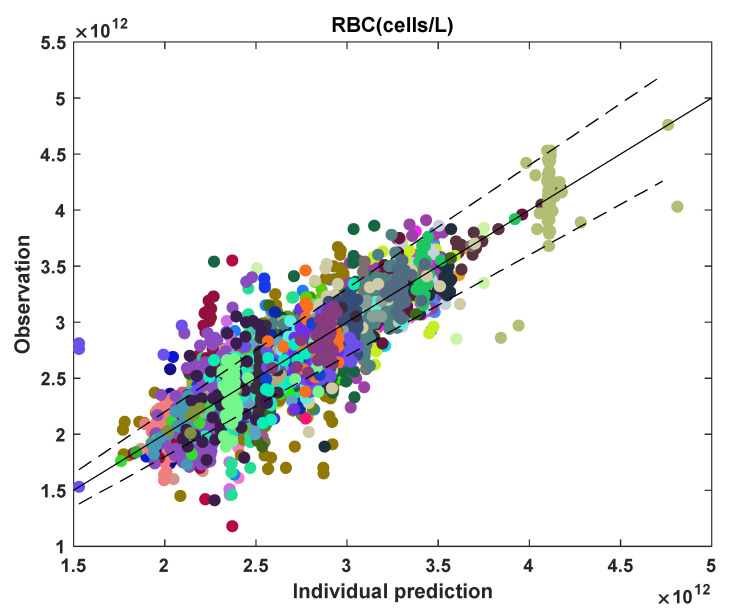
Observation versus individual prediction of RBC for all data points across the population. Each color marks an individual participant. The solid line represents y = x; the dashed lines represent 10% error.

Further, the observation versus individual prediction plot for all the RBC data is displayed in Figure 14. For most of the participants, the model fit lies within 10% error of the y = x line. For ~18% of the data points, the model fits lie outside the 10% error region. Here, for participants with clear myelosuppression trends, the model fits well to the data. For fluctuating RBC, the model does not capture the trends in data well. Table 3 gives the RBC and MCV model estimated parameter statistics.

### 3.3. ANC Model

**Figure 15:**
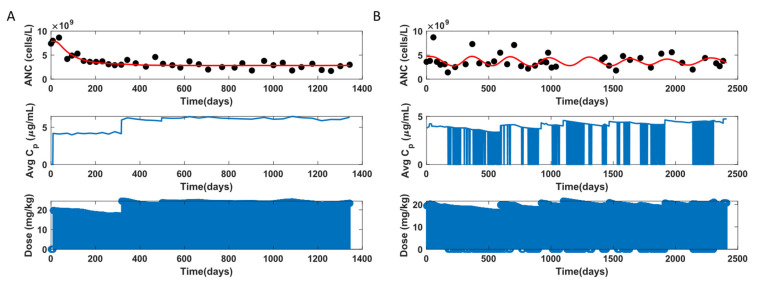
ANC model fit to data for two representative participants. (**A**): Adherent, (**B**): Non-adherent.

The ANC model was fit to the clinical data of 83 individuals, omitting two participants due to insufficient data points. The ANC of individuals with SCD is usually elevated, as leukocytes are recruited to adhere to the vessel wall and play a role in vaso-occlusion [60]. In Figure 15A, the myelosuppression effect is evident, where ANC decreases from 8000 cells/μL and reaches a steady state between 2000–4000 cells/μL, which is the ideal target range for ANC. The above is an example of an adherent participant, as apparent from the ${\bar{C}}_{p}$ profile here, and the model fits the data well in this case. Figure 15B displays a non-adherent participant, where there are multiple non-adherent days. When the data only exhibits fluctuations without any myelosuppression trend, the model mimics the trend with a periodic solution, as shown here, or a stable steady-state solution (not shown here). The model does not perform well when the neutrophil count fluctuates but does not show any periodic behavior.

**Figure 16:**
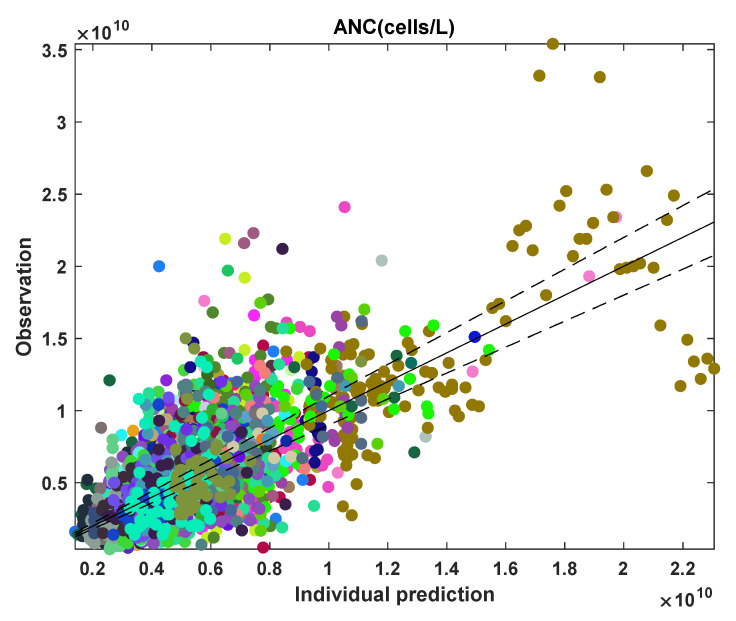
Observation versus individual prediction for absolute neutrophil count (ANC) of all data points across the population, with each color marking an individual participant. The solid line represents y = x; the dashed lines represent 10% error.

Figure 16 shows an observation versus individual prediction for all data points of the subjects. Many data points, ~80%, fall outside the 10% error for individual predictions because the neutrophil data are highly fluctuating. The model fits well to the data where clear myelosuppression trends in ANC are observed. However, the model performs poorly when there are high ANC fluctuations. Table 4 lists the statistics for the leukopoiesis model parameter estimates.

### 3.4. HbF Model

**Figure 17:**
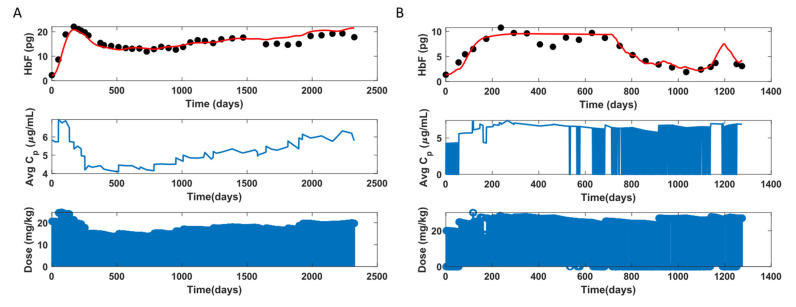
HbF model fit of two representative participants. (**A**): Adherent, (**B**): Non-adherent.

The HbF model was fit to 81 individual participants’ data, leaving 4 individuals out due to insufficient data points. The HbF model performance for the HbF participant data is demonstrated in Figure 17. The participant in Figure 17A is adherent, as seen from the ${\bar{C}}_{p}$ profile where the participant is not missing a dose. The ${\bar{C}}_{p}$ increases and decreases and then rises again. The initial increase in HbF is due to HU treatment initiation, and further changes in HbF follow a trend similar to that of the ${\bar{C}}_{p}$. The model fits this individual very well. The participant in Figure 17B is non-adherent at times, also shown in Figure 11B. Similarly to the MCV profile, the drop in the HbF was captured when the dose input contained the missing dose information. So, the HbF model can fit adherent and non-adherent participants conditionally, given that the dosing profile accurately describes non-adherence.

**Figure 18:**
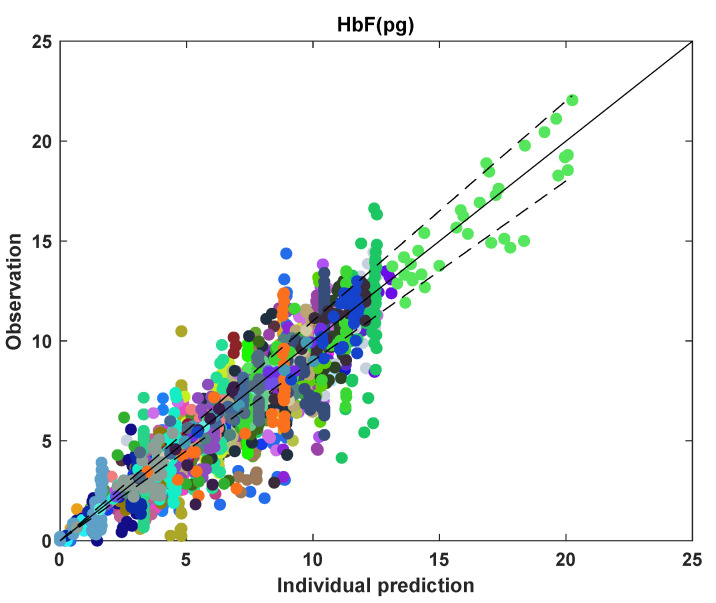
Goodness-of-fit plot showing mean cell fetal hemoglobin for all data points with each color marking an individual. The solid line represents y = x; the dashed lines represent 10% error.

In Figure 18, it is seen that many data points fall outside the 10% error for individual predictions, with ~54% of data points outside this region. For some participants with data points outside the 10% error region, the individual predictions were higher than the observed values, indicating overprediction. This might happen when the participant starts missing the dose after HbF reaches its maximum saturation value. The lower clinical value of HbF indicates that the participant might have missed the dose. Still, if the dosing information does not contain those missing doses, the model will predict a higher level of HbF. Moreover, for many participants, the number of data points for HbF was scarce and lower than the number of data points for MCV and other variables. The scarcity of HbF data can lead to the model not representing the non-adherence in the dosing profile well. Table 5 summarizes the parameter estimates for the HbF model.

## 4. Discussion

During HU treatment, while HU is cleared from the body within 24 h, it takes weeks to see its effect on the biomarker levels. Existing models have focused mainly on predicting the HU PK and optimizing the dose based on the PK parameters; only a few studies have explored the relationship between PK and PD models. In this work, the focus was to build an integrated model to explain the substantial variability in the PK-PD of individuals with SCD receiving HU treatment. Our integrated PK-PD model can be used to quantitatively describe the treatment mechanism and be applied for planning dosing regimens.

The PK model gives reasonable accuracy by calculating and fitting the clinical PK parameter values obtained from NCA. Since the data that are matched are AUC, ${\mathrm{AUC}}_{\infty }$, which are integrated values of $C_{p}$ over time, it is possible that even when the model is able to match AUC and other integrated clinical PK parameters, the model might not exactly replicate the actual time course of $C_{p}$. This can cause model identifiability issues, as multiple parameter sets can estimate clinical PK parameters close to the data. In this case, matching $C_{\max}$ and $T_{\max}$ helps in making sure that in the model, the peak concentration occurs at the exact timepoint and value as the data provided. Therefore, having $C_{p}$ vs. time data would help us in improving PK model accuracy. Another challenge was that the everyday dosing information was not available. The daily dose was computed from the pharmacy records available for individual participants. While calculating everyday dose, it was assumed that the total dose remained constant in between visits. The ${\bar{C}}_{p}$ was determined daily by simulating the PK model with the computed daily dose. This approach has limitations in that the PK model assumes the parameter to remain constant with the participant’s age or with changes in other variables. However, the fact that these participants were pediatric made it more complicated, as they underwent several physical changes such as changes in height, weight, or physiological or anatomical changes, thus leading to the possibility that the absorption, distribution, metabolism, and excretion (ADME) of the drug might change. The change in ADME will dictate the change in PK model parameters.

Among the various PD clinical variables that were modeled, the MCV model performed well for most participants because of low variability. The MCV model was adapted from the work of Jayachandran et al., where the drug 6-mercaptopurine, similar to HU, increased MCV levels after initiation [44]. The HbF model performed well for those participants whose dosing information was likely accurate. The model fit well for participants who fell into the two categories: adherent participants and non-adherent participants, where non-adherence was seen in both the drug input and the data. However, when the drug input does not contain non-adherence information, but the data for HbF or MCV indicate non-adherence, a discrepancy occurs between the model and the data. Overall, these models help in describing the mechanism of HU in SCD. Further, some participants received blood transfusions. Separating such cases from non-adherence and incorporating and modeling blood transfusion will help improve the fit for the individuals when they receive a transfusion.

In the HbF model, one of the assumptions is that the basal rate of HbF production remains constant with age, but this need not be the case, especially if the participant is starting on HU before 1 year of age. Considering the dependency of basal HbF production rate on age might help improve the fits for participants under 1 year of age when they are initiated on HU treatment. Certain participants with a higher basal rate of production of HbF might indicate the presence of HPFH. Another assumption of HbF is that every RBC makes the same amount of HbF, but it is seen that certain RBCs produce a higher amount of HbF than others. Few studies have captured the distribution of F cell percentage [61]. A similar assumption was made for the MCV model, where every RBC was assumed to have the same cell volume.

The RBC and ANC data showed either a clear myelosuppression trend or a lack of any trend. When there was a clear myelosuppression trend, the model performed reasonably well. In contrast, when there was a lack of a clear trend, the model fit the data with a periodic solution if the data exhibited some periodicity. The model matched the data with a straight line or curve if the data did not show any periodicity. The ANC data of participants fluctuated. There are various reasons for neutrophils to demonstrate this behavior. The fluctuations in ANC can be due to disease-related complications and treatment-related issues as well as regular physiological changes. Additionally, the increase in ANC can be due to non-adherence and common infections such as cold, cough, fever, and flu. The model presented in this work did not consider such fluctuations, so it could not capture the participants who showed these fluctuations.

The models developed here could pave the path for individualized treatment of individuals affected with SCD quantitatively, which could help save time and effort for clinicians as well as participants. The models formulated in this work could be used to determine the individual trajectory of key biomarkers as well as keep the blood cell counts within the target range and determine the optimal dose in a short time span compared to the time spent in the clinic to determine the MTD. The multiple responses of individuals with SCD demand a thorough analysis and monitoring of participants’ biomarkers, blood cell counts, and metabolites. When a patient is unresponsive, the interesting thing to explore will be whether the treatment is not very effective due to PK-related effects such as the lower activity of the transporting proteins or PD-related effects such as lower HbF synthesis. There appears to be a need to track non-adherence more rigorously so that model predictions can more closely correlate with clinical measurements.

## Figures and Tables

**Table 1:** Summary of participants with SCD characteristics.

Demographics			
Starting age of HU treatment (years)	10.12 (4.72) Mean (SD)		
Male/Female	54/31		
**Clinical PK parameters**	**Mean (SD)**		
AUC (µg∙h/mL)	85.79 (19.64)		
AUC∞ (µg∙h/mL)	92.98 (23.37)		
MRT∞ (h)	3.17 (0.78)		
Tmax (h)	0.82 (0.47)		
Cmax (µg/mL)	26.13 (6.83)		
λz (h^−1^)	0.65 (0.23)		
**PD variables**	**At baseline** **Mean (SD)**	**After 6 months of treatment** **Mean (SD)**	**After 12 months of treatment** **Mean (SD)**
MCV (fL)	85.34 (6.958)	103.1 (10.46)	104.1 (10.06)
MCH (pg)	29.76 (2.842)	35.51 (3.866)	36 (3.876)
HCT (%)	23.46 (3.649)	26.9 (4.102)	27.12 (3.858)
Hb (g/dL)	8.151 (1.143)	9.246 (1.332)	9.364 (1.279)
HbF (%)	8.004 (5.978)	20.54 (8.854)	21.65 (9.129)
HbS (%)	72.92 (20.09)	68.65 (9.441)	67.34 (11.99)
ANC (cells/µL)	6814 (3384)	4449 (2779)	4007 (1891)
$\mathrm{WBC}\;(\times 10^{3}$ cells/µL)	13.51 (4.981)	9.114 (3.273)	8.418 (2.864)
$\mathrm{ARC}\;(\times 10^{6}$ cells/µL)	0.2711 (0.0958)	0.1563 (0.0840)	0.1522 (0.0615)
$\mathrm{RBC}\;(\times 10^{6}$ cells/µL)	2.777 (0.5517)	2.636 (0.4918)	2.628 (0.4709)

Note: SD, standard deviation; HU, hydroxyurea; AUC, area under the concentration–time curve from time 0 to the last time plasma concentration was measured; AUC_∞_, area under the concentration–time curve when extrapolated to time ∞; MRT_∞_, mean residence time; T_max_, the time point at which the maximum plasma concentration is observed; C_max,_ maximum observed plasma concentration; λ_z_, terminal elimination rate constant; MCV, mean cell volume; MCH, mean cell hemoglobin; HCT, hematocrit; Hb, hemoglobin; HbF, fetal hemoglobin; HbS, sickle hemoglobin; ANC, absolute neutrophil count; WBC, white blood cell; ARC, absolute reticulocyte count; RBC, red blood cell.

**Table 2:** Pharmacokinetic model parameter values for HUSTLE participants.

Parameter	Mean (SD)
F	0.12 (0.04)
$k_{tr}$ (h^−1^)	5.02 (2.61)
$N_{t}$	1.14 (1.08)
$k_{e}$ (h^−1^)	0.54 (0.26)
$V_{p}$ (L)	1.77 (0.88)

**Table 3:** RBC and MCV model parameter estimates for HUSTLE participants.

Parameter	Median	1st Quartile	3rd Quartile
$k_{pemax}$ (cells/L/day)	2.10 × 10^12^	1.30 × 10^11^	1.90 × 10^14^
$\Psi_{e}$ (cells/L)	2.20 × 10^11^	7.10 × 10^10^	1.10 × 10^12^
$\gamma_{e}$	1.6	0.52	3.3
$k_{dse,max}$ (1/day)	0.17	0.02	0.71
$K_{dse,50}$ (µM)	0.43	0.01	4.4
$k_{te}$ (1/day)	0.2	0.05	0.37
$k_{de,max}$ (1/day)	0.03	0.01	0.06
$K_{de,50}$ (µM)	310	62	860
α (fL/µM)	0.37	0.23	0.51

**Table 4:** Leukopoiesis model parameter estimates for HUSTLE participants.

Parameter	Median	1st Quartile	3rd Quartile
$k_{plmax}$ (cells/L/day)	1.10 × 10^12^	3.30 × 10^10^	1.80 × 10^14^
$\Psi_{l}$ (cells/L)	4.70 × 10^8^	1.50 × 10^8^	1.20 × 10^9^
$\gamma_{l}$	3.5	2	4.3
$k_{dsl,max}$ (1/day)	0.16	0.04	1.2
$K_{dsl,50}$ (µM)	0.3	0.01	30
$k_{tl}$ (1/day)	0.09	0.03	0.32
$k_{dl}$ (1/day)	0.13	0.03	0.45

**Table 5:** Fetal hemoglobin model parameter estimates for HUSTLE participant data.

Parameter	Median	1st Quartile	3rd Quartile
$k_{met}$ (µM/day)	1.2	0.05	3.5
$K_{met}$ (µM)	27	4	110
$k_{di}$ (1/day)	0.03	0.01	0.06
$k_{bf}$ (pg/day)	0.16	0.04	0.64
$k_{af}$ (pg/day)	1.5	0.3	4.3
$K_{af}$ (µM)	13	2.2	45
n	3.2	1.6	4.5
$k_{df}$ (1/day)	0.07	0.02	0.3

## Data Availability

Data are available upon request from the authors.

## Appendix A

*Mean Cell Fetal Hemoglobin Calculation*

There are two options to compute $F_{m}$:

1. *F_m_* (pg) is calculated by multiplying HbF (%) by mean cell hemoglobin, MCH (pg), as shown below:
  (A1) $$F_{m}\;\left(\mathrm{pg}\right)=\frac{HbF\;\left(\%\right)}{100}\times MCH\left(\mathrm{pg}\right)$$
2. *F_m_* (pg) is also calculated by multiplying HbF (%) by hemoglobin, Hb (g/dL), dividing by hematocrit, HCT (%), and multiplying by MCV (fL).
  (A2) $$F_{m}\;\left(\mathrm{pg}\right)=\frac{HbF\;\left(\%\right)}{100}\times \frac{Hb\left(\frac{g}{\mathrm{dL}}\right)}{HCT\;\left(\%\right)}\times \frac{MCV\left(\mathrm{fL}\right)}{100}$$

Both methods give approximately the same $F_{m}$, so for all the calculations, $F_{m}$ determined with Equation (A1) was used.

*PK Compartment Modeling*

In this transit compartment model, a simple mass balance equation describing the rate of change in drug in different compartments is set up using mass-action kinetics. The drug amount in different compartments is given by [29,39],

(A3) $$\frac{da_{0}}{dt}=-k_{tr}a_{0};\;a_{0}\left(t=0\right)=FD\;\frac{da_{i}}{dt}=k_{tr}\left(a_{i}-a_{i-1}\right);a_{i}\left(t=0\right)=0;\;for\;i=\left\{1,2,..,\;N_{t}\right\}$$

where $\left(N_{t}+1\right)$ is the number of transit compartments, $a_{0}$ and $a_{i}$ are the drug amounts in the 0 and ith compartments, respectively, $k_{tr}$ is the transit rate constant, *F* is the drug bioavailability, D is the drug input.

Solving Equation (A3) analytically, the drug amount in the final compartment in the gut, $a_{N_{t}}$, is given by,

(A4) $$a_{N_{t}}=FD\frac{{\left(k_{tr}t\right)}^{N_{t}}e^{-k_{tr}t}}{N_{t}!}$$

*Average Drug Concentration Calculation*

The ${\bar{C}}_{p}$ is calculated using the formula below:

(A5) $${\bar{C}}_{p}=\frac{\int_{t_{0}}^{t_{1}}C_{p}dt}{\left(t_{1}-t_{0}\right)}$$

*MCV Calculation*

The MCV is given by,

(A6) $$V_{m}=\frac{V_{TOT}}{N_{e}}$$

The rate of change in MCV is obtained from the above equation as follows:

(A7) $$\frac{dV_{m}}{dt}=\frac{d}{dt}\left(\frac{V_{TOT}}{N_{e}}\right)=\frac{1}{N_{e}}\frac{dV_{TOT}}{dt}-\frac{V_{m}}{N_{e}}\frac{dN_{e}}{dt}$$

After substituting Equations (9) and (13) in (A7), the rate of change in MCV is given by,

(A8) $$\frac{dV_{m}}{dt}=\frac{\left(\alpha {\bar{C}}_{p}+V_{m0}-V_{m}\right)k_{te}N_{e3}}{N_{e}}$$
